# Clinical usefulness of urinary liver-type fatty-acid-binding protein as a perioperative marker of acute kidney injury in patients undergoing endovascular or open-abdominal aortic aneurysm repair

**DOI:** 10.1007/s00540-015-2095-8

**Published:** 2015-11-19

**Authors:** Yumi Obata, Atsuko Kamijo-Ikemori, Daisuke Ichikawa, Takeshi Sugaya, Kenjiro Kimura, Yugo Shibagaki, Takeshi Tateda

**Affiliations:** Department of Anesthesiology, St. Marianna University School of Medicine, 2-16-1 Sugao, Miyamae-Ku, Kawasaki, 216-8511 Japan; Division of Nephrology and Hypertension, Department of Internal Medicine, St. Marianna University School of Medicine, Kanagawa, Japan; Tokyo Takanawa Hospital, Tokyo, Japan

**Keywords:** Urinary liver-type fatty-acid-binding protein, Acute kidney injury, Abdominal aortic repair

## Abstract

**Purpose:**

Acute kidney injury (AKI) is common after cardiovascular surgery and is usually diagnosed on the basis of the serum creatinine (SCr) level and urinary output. However, SCr is of low sensitivity in patients with poor renal function. Because urinary liver-type fatty-acid-binding protein (L-FABP) reflects renal tubular injury, we evaluated whether perioperative changes in urinary L-FABP predict AKI in the context of abdominal aortic repair.

**Methods:**

Study participants were 95 patients who underwent endovascular abdominal aortic aneurysm repair (EVAR) and 42 who underwent open repair. We obtained urine samples before surgery, after anesthesia induction, upon stent placement, before aortic cross-clamping (AXC), 1 and 2 h after AXC, at the end of surgery, 4 h after surgery, and on postoperative days (PODs) 1, 2, and 3, for measurement of L-FABP. We obtained serum samples before surgery, immediately after surgery, and on PODs 1, 2, and 3, for measurement of SCr. We also plotted receiver-operating characteristic (ROC) curves to identify cutoff laboratory values for predicting the onset of AKI.

**Results:**

With EVAR, urinary L-FABP was significantly increased 4 h after the procedure (*P* = 0.014). With open repair, urinary L-FABP increased significantly to its maximum by 2 h after AXC (*P* = 0.007). With AKI, SCr significantly increased (*P* < 0.001, *P* = 0.001) by POD 2. ROC analysis showed urinary L-FABP to be more sensitive than SCr for early detection of AKI.

**Conclusion:**

Urinary L-FABP appears to be a sensitive biomarker of AKI in patients undergoing abdominal aortic repair.

## Introduction

Acute kidney injury (AKI) is common after cardiovascular surgery, sometimes requiring postoperative hemodialysis, and is associated with increased morbidity and mortality [1]. AKI occurs after abdominal aortic aneurysm repair surgery in >30 % of cases and leads to a prolonged hospital stay [2]. Both open abdominal aortic aneurysm repair and infrarenal aortic cross-clamping (AXC) decrease renal blood flow and increase renal vascular resistance. These changes, which indicate diminished global perfusion with redistribution of renal blood flow toward the cortical compartment, persist for at least 1 h after release of the aortic clamp [3–5]. Infrarenal AXC produces profound and sustained alterations in renal hemodynamics and may be particularly harmful in patients with impaired renal function or when it is prolonged [3].

Endovascular abdominal aortic aneurysm repair (EVAR), performed with intra-arterial contrast enhancement, has become an important treatment for infrarenal abdominal aortic aneurysm. Although prospective studies have shown better renal outcomes with EVAR than with open repair, the long-term durability and safety of stent grafting remain unclear [6–8]. EVAR requires intra-arterial administration of contrast medium, which can impair renal function and even lead to end-stage renal disease [9–11]. Although the incidence of contrast-induced AKI is low (2 %) in the general population, it is high (5–10 %) in patients at risk for kidney disease, such as those with cardiovascular disease [12, 13]. Early diagnosis and treatment of AKI may be essential for improved perioperative renal outcomes after both EVAR and open repair. Biomarkers are used effectively in the diagnosis of AKI, with assay of serum creatinine (SCr) being the gold standard. However, SCr is of low sensitivity in patients with poor renal function or low muscle mass [14, 15]. Several new biomarkers of kidney injury have been investigated, both experimentally and clinically, with liver-type fatty-acid-binding protein (L-FABP) being a promising candidate [16]; it is recognized as the most useful alternative biomarker of kidney injury. Matsui et al. described urinary L-FABP as an early predictor of AKI after cardiac surgery [17]. In addition, Kamijo et al. showed urinary L-FABP to be an excellent biomarker for clinical prediction and monitoring of renal disease [18]. Several studies have shown the usefulness of urinary L-FABP for the detection of AKI after cardiac surgery [19] and contrast-induced nephropathy [20].

We conducted a prospective study with two aims: (1) to evaluate the perioperative changes in urinary L-FABP that occur with EVAR and open abdominal aortic aneurysm repair, and (2) to examine the usefulness of urinary L-FABP for predicting AKI after either type of abdominal aortic repair.

## Methods

The study protocol was approved by the Institutional Review Board of St. Marianna University School of Medicine (No 1966), Kawasaki, Japan, and registered University Hospital Medical Information Network (UMIN) Clinical Data Registry (ID 000006584). Written informed consent was obtained from all patients enrolled in the study.

### Study design

We conducted a two-part prospective study in a university hospital setting: one part to investigate AKI associated with EVAR and the other to investigate AKI associated with open repair. Consecutive patients scheduled for EVAR (*n* = 95) or open repair (*n* = 42) between October 2011 and June 2015 were enrolled. Each patient’s surgeon chose between EVAR and open repair by considering the patient’s age and the type of abdominal aortic aneurysm in the absence of any concern regarding the patient’s tolerance for study procedures. Excluded from the study were patients undergoing dialysis or requiring emergency surgery.

### EVAR study protocol

#### Anesthesia

No premedication or epidural anesthesia was administered to any patient in the EVAR group. General anesthesia was induced with remifentanil and propofol. Tracheal intubation was facilitated with rocuronium, and general anesthesia was maintained with sevoflurane in an air–oxygen mixture and remifentanil.

#### Sample collection

Urine samples (10 ml) were obtained before surgery, after anesthesia induction, upon stent placement, at the end of surgery, 4 h after surgery, and on postoperative days (PODs) 1, 2, and 3 for measurement of urinary L-FABP and urinary albumin. Urine samples were centrifuged at 1000 g for 5 min at 4 °C and stored at −80 °C until analysis. In addition, serum samples were obtained before surgery, immediately after surgery, and on PODs 1, 2, and 3 to measure serum creatinine (SCr).

### Open-repair study protocol

#### Anesthesia

No premedication was administered to any patient in the open-repair group. All patients in this group received epidural anesthesia before the surgery. An epidural catheter was inserted via the Th9/10, Th10/11, or Th11/12 intervertebral space. General anesthesia was induced with remifentanil and propofol. Tracheal intubation was facilitated with rocuronium. Anesthesia was maintained with sevoflurane in an oxygen–air mixture and remifentanil. Levobupivacaine (0.125 or 0.25 %) was administered via epidural catheter during the surgery. Dopamine, prostaglandin E_1_, and carperitide were infused continuously during surgery.

#### Sample collection

Urine samples (10 ml) were obtained before surgery, after anesthesia induction, before aortic cross-clamping (AXC), 1 and 2 h after AXC, at the end of surgery, 4 h after surgery, and on PODs 1, 2, and 3 for measurement of urinary L-FABP and urinary albumin. These urine samples were prepared and stored as described above. In addition, serum samples were obtained before surgery, immediately after surgery, and on PODs 1, 2, and 3 to measure SCr.

### Clinical monitoring of EVAR and open-repair patients

During the first 48 h postoperative period, we monitored patients for AKI as defined according to AKI network criteria [21]. We also monitored patients’ estimated glomerular filtration rate (eGFR) at the start of this study, which was calculated according to the Japanese-coefficient-modified Chronic Kidney Disease Epidemiology Collaboration equation: ${\text{eGFR}} = 1 9 4 \times \left( {\text{creatinine}} \right)^{ - 1.0 9 4} \times \left( {\text{age}} \right)^{ - 0. 2 8 7} \times \left( {0. 7 3 9 {\text{ if female}}} \right)$ [22].

### Assay of urinary L-FABP, urinary albumin, and SCr

Urinary L-FABP levels were determined by enzyme-linked immunosorbent assay (ELISA) with use of the human L-FABP ELISA kit (CMIC, Tokyo, Japan). Urinary albumin was measured by immunonephelometry. SCr was measured by an enzymatic method.

### Statistical analyses

Study variables are expressed as median [interquartile range (IQR)]. Between-group (AKI vs. non-AKI; EVAR vs. open repair) differences were analyzed using the Mann–Whitney *U* test or chi-square test, as appropriate. One-way analysis of variance (ANOVA), followed by Dunnett’s post hoc test, was used for multiple comparisons. Receiver-operating characteristic curves (ROCs) were plotted to identify cutoff laboratory values for predicting AKI onset. Univariate analysis was used to select the clinical risk factor for the occurrence of AKI and the characteristics showing a significant difference between AKI and non-AKI groups. Following univariate analysis, significant unadjusted predictors with *P* < 0.05 were used in a multivariate logistic regression analysis. Multivariate logistic regression analysis was performed using a forward selection method. All statistical analyses were performed with IBM SPSS Statistics, version 21.0 (IBM, Tokyo, Japan). *P* < 0.05 was considered significant for all analyses.

## Results

### EVAR study

**Fig. 1:**
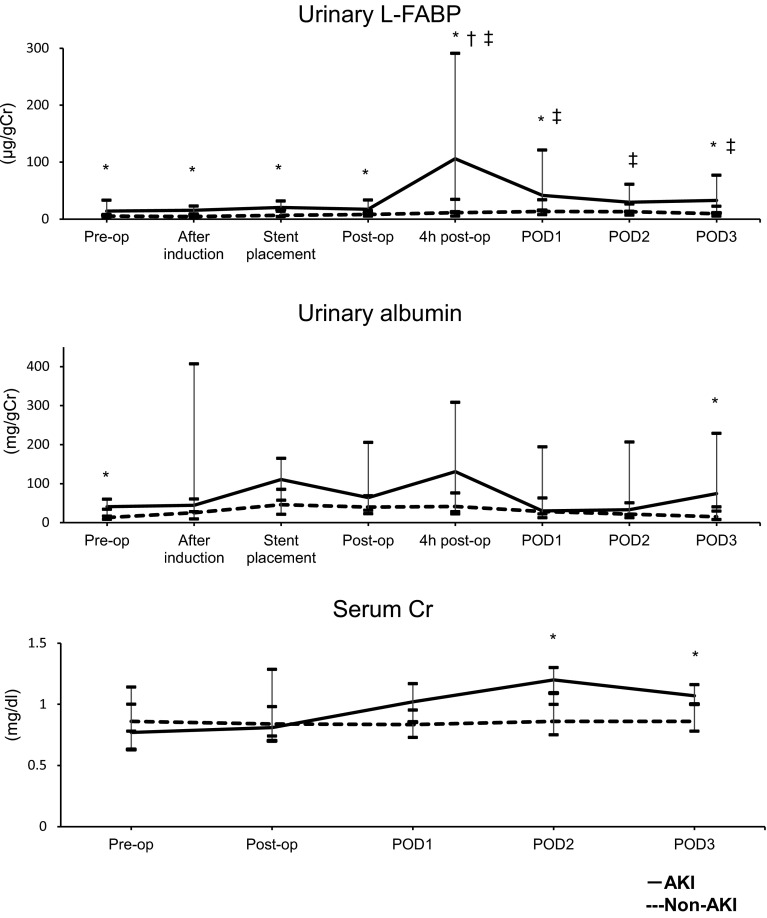
Changes in urinary L-FABP, urinary albumin, and SCr in EVAR. Acute kidney injury (AKI; *solid line*) and non-AKI groups (*dashed line*). *L*-*FABP* liver-type fatty acid-binding protein, *SCr* serum creatinine, *EVAR* endovascular aneurysm repair, *Pre*-*op* preoperative value, *After induction* value after induction of anesthesia, *Post*-*op* immediate postoperative value, *4* *h post*-*op* value 4 h after the operation, *POD* postoperative day. *P < 0.05 vs. non-AKI group at the same time point; ^†^ *P* < 0.05 vs. respective preoperative level in the same (AKI) group; ^‡^ *P* < 0.05 vs. respective preoperative level in the same (non-AKI) group

AKI developed postoperatively in nine (9.5 %) of the 95 patients enrolled in the EVAR study: stage 1 AKI in eight patients and stage 2 AKI in one patient. None required postoperative renal replacement therapy (RRT). Body weight (*P* = 0.03) and body mass index (BMI) (*P* = 0.001) were significantly lower, diabetes mellitus (*P* = 0.049) and nonsteroidal anti-inflammatory drug (NSAID) use (*P* = 0.002) were significantly more prevalent, and duration of anesthesia (*P* = 0.046) and length of hospital stay (*P* = 0.005) were significantly longer in the AKI group than in the non-AKI group (Table 1). Before surgery, after anesthesia induction, and upon stent placement, urinary L-FABP level was high and was significantly increased (*P* = 0.014) in the AKI group at 4 h after surgery; it decreased over the three PODs (Fig. 1).

**Table 1:** Patient characteristics and clinical outcomes in EVAR study

	Non–AKI (*n* = 86)	AKI (*n* = 9)	*P* value
Sex (M/F)	70/16	8/1	
Age (years)	78 (73–83)	78 (75–82)	0.949
Body weight (kg)	60 (53–68)	51 (48–61)	0.030
BMI (kg/m^2^)	23 (21–25)	20 (17–21)	0.001
ASA status (II/III)	67/19	5/4	0.138
Comorbidity, *n* (%)			
Diabetes mellitus	9 (10)	3(33)	0.049
Hypertension	69 (80)	9 (100)	0.141
Ischemic heart disease	33 (38)	3 (33)	0.787
Chronic kidney disease	18 (21)	3 (33)	0.394
Concomitant medications, *n* (%)			
Ca inhibitor	51 (59)	7 (78)	0.297
ACE inhibitor/ARB	38 (44)	4 (44)	0.988
Statin	31 (36)	2 (22)	0.407
Diuretic	8 (9)	1 (11)	0.860
NSAID	0 (0)	1 (11)	0.002
Smoking history	41 (48)	5 (56)	0.802
Preoperative urinary L-FABP (μg/g Cr)	5 (3.3–7.9)	14.1 (6.5–32.9)	0.002
Preoperative urinary albumin (mg/g Cr)	13.0 (7.9–33.6)	40.7 (16.4–59.9)	0.016
Preoperative SCr (mg/dl)	0.86 (0.77–1.01)	0.77 (0.63–1.14)	0.644
Preoperative eGFR^a^ (ml/min)	63.1 (51.1–69.1)	57.5 (48.7–87.3)	0.854
General anesthesia	86	9	
General anesthesia with epidural	0	0	
Duration of anesthesia (min)	208 (185–234)	225 (220–290)	0.046
Duration of surgery (min)	128 (105–155)	145 (133–210)	0.069
Fluids infusion (ml)	1500 (1213–1800)	1650 (1300–2000)	0.337
Estimated blood loss (ml)	78 (39–143)	114 (76–148)	0.230
Contrast media (ml)	118 (100–160)	143 (116–226)	0.138
Operative and postoperative details			
Mechanical ventilation, *n* (%)	0 (0)	0 (0)	
Length of hospital stay (days)	13 (12–14)	17 (14–23)	0.005
RRT required upon discharge	0 (0)	0 (0)	
In-hospital death	0 (0)	0 (0)	

Data are expressed as median (interquartile range) or number (%)

*EVAR* endovascular aortic repair, *AKI* acute kidney injury, *BMI* body mass index, *ASA* American Society of Anesthesiologists*, ACE inhibitor* angiotensin-converting enzyme inhibitor, *ARB* angiotensin II receptor blocker, *NSAID* nonsteroidal anti-inflammatory drug,* Ca* calcium channel, * Cr* creatinine, *SCr* serum creatinine, *eGFR* estimated glomerular filtration rate, *RRT* renal replacement therapy

^a^eGFR was calculated according to the Japanese coefficient-modified Chronic Kidney Disease Epidemiology Collaboration equation: eGFR = 194 × (creatinine)^−1.094^ × (age)^−0.287^ × (0.739 if female)^22^

SCr and urinary albumin did not change in the non-AKI group during the perioperative period; however, in the AKI group, it increased significantly on PODs 2 and 3 (*P* = 0.000 and *P* = 0.011, respectively; Fig. 1). Multivariate logistic regression analysis performed for both groups showed preoperative urinary L-FABP level to be a predictor of postoperative AKI [odds ratio (OR) 6.76; confidence interval (CI) 1.76–25.94, *P* = 0.005; Table 2]. BMI was also shown to be a predictor factor. The cutoff preoperative urinary L-FABP level was 9.0 μg/g Cr (Table 3).

**Table 2:** Multivariate logistic regression analyses for AKI in the EVAR group

Variable	Multivariate analysis		
	OR	95 % CI	*P* value
BMI	0.51	0.31–0.84	0.008
Diabetes mellitus	1.46	0.89–2.39	0.138
NSAID	5.85	0.00–0.00	1.000
Length of hospital stay	1.13	0.76–1.67	0.553
Urinary L-FABP pre-operation	6.76	1.76–25.94	0.005
Urinary L-FABP after induction	0.65	0.05–9.19	0.746
Urinary L-FABP stent placement	10.6	0.59–190.9	0.109
Urinary L-FABP 4 h postoperation	0.96	0.24–3.83	0.957
SCr POD2	115.9	0.72–18,548.3	0.066

*AKI* acute kidney injury, *EVAR* endovascular aneurysm repair, *OR* odds ratio, *CI* confidence interval, *BMI* body mass index, *NSAID* nonsteroidal anti-inflammatory drug, *L*-*FABP* liver-type fatty-acid-binding protein, *SCr* serum creatinine, *POD* postoperative day

**Table 3:** Urinary L-FABP levels predictive of AKI in the EVAR study

Time point	Cutoff value (μg/g Cr)	Sensitivity	Specificity	PPV	NPV
Preoperation	9.0	0.67	0.82	0.63	0.85
After anesthesia induction	5.9	0.89	0.61	0.51	0.93
At stent placement	16.3	0.78	0.80	0.64	0.89
Postoperation	9.2	0.89	0.57	0.48	0.92
4 h postoperation	87.8	0.56	0.92	0.76	0.82
POD1	68.1	0.44	0.87	0.63	0.78
POD2	28.1	0.56	0.74	0.48	0.79
POD3	62.3	0.44	0.92	0.71	0.79

*L*-*FABP* liver-type fatty-acid-binding protein, *AKI* acute kidney injury, *EVAR* endovascular aneurysm repair, *PPV* positive predictive value, *NPV* negative predictive value, *POD* postoperative day,* Cr* creatinine

### ROC analysis

The biomarker with the largest area under the curve (AUC) for predicting AKI onset was urinary L-FABP at the following time points: before surgery, after anesthesia induction, upon stent placement, and 4 h after surgery; AUCs were 0.83, 0.81, 0.79, and 0.75, respectively (Table 4). Urinary L-FABP cutoff values at different time points for EVAR are shown in Table 3.

**Table 4:** AUC vs. time in the EVAR group

	Preoperation	After induction	Stent placement	Postoperation	4-h postoperation	P0D1	P0D2	P0D3
Urinary l-FABP	0.83 (0.69–0.96)	0.81 (0.69–0.93)	0.79 (0.64–0.95)	0.72 (0.54–0.90)	0.75 (0.57–0.94)	0.70 (0.52–0.88)	0.69 (0.50–0.87)	0.71 (0.52–0.90)
Urinary albumin	0.72 (0.57–0.87)	0.67 (0.48–0.86)	0.71 (0.52–0.90)	0.63 (0.42–0.84)	0.65 (0.42–0.88)	0.62 (0.43–0.82)	0.64 (0.45–0.84)	0.76 (0.57–0.95)
SCr	0.45 (0.18–0.72)	–	–	0.54 (0.28–0.79)	–	0.69 (0.50–0.88)	0.84 (0.74–0.94)	0.73 (0.57–0.89)

Data are given as AUC (95% confidence interval)

*AUC* area under the curve, *EVAR* endovascular aortic repair, *After induction* after induction of anesthesia, *Postoperation* immediately after surgery, *POD* postoperative day, *L-FABP* liver-type fatty-acid-binding protein,* SCr* serum creatinine

### Open-repair study

AKI developed postoperatively in 13 (31.0 %) of the 42 patients enrolled in the open-repair study and was stage 1 in all of them. No patient required postoperative RRT. Male sex (*P* = 0.000) was more prevalent, age (*P* = 0.025) was greater, and preoperative ischemic heart disease (*P* = 0.016) more prevalent among open-repair patients in the AKI than in the non-AKI group (Table 5).

**Table 5:** Patient characteristics and clinical outcomes in the open-repair study

	Non–AKI group (*n* = 29)	AKI group (*n* = 13)	*P* value
Sex (M/F)	26/3	12/1	<0.001
Age (years)	67 (64–72)	72 (69–78)	0.025
Body weight (kg)	61 (56–74)	68 (64–69)	0.187
BMI (kg/m^2^)	23 (21–25)	24 (24–26)	0.094
ASA status (II/III)	21/8	7/6	0.486
Comorbidity, *n* (%)			
Diabetes mellitus	2 (7)	3 (23)	0.134
Hypertension	24 (92)	12 (92)	0.414
Ischemic heart disease	13 (45)	11 (85)	0.016
Chronic kidney disease	7 (24)	5 (38)	0.342
Concomitant medications, *n* (%)			
Ca inhibitor	17 (59)	8 (62)	0.859
ACE inhibitor/ARB	17 (59)	8 (62)	0.859
Statin	14 (48)	5 (38)	0.555
Diuretic	2 (7)	2 (15)	0.386
NSAID	1 (3)	0 (0)	0.498
Smoking history	16 (55)	7 (54)	0.936
Preoperative urinary L-FABP (μg/g Cr)	4.1 (2.3–7.0)	4.4 (2.9–11.9)	0.863
Preoperative urinary albumin (mg/g Cr)	9.9 (6.4–13.8)	20 (8.3–69.3)	0.128
Preoperative SCr (mg/dl)	0.85 (0.75–1.06)	0.92 (0.85–1.31)	0.268
Preoperative eGFR^a^ (ml/min)	67.3 (52.7–77.9)	62.3 (44.8–68.7)	0.196
General anesthesia	0	0	
General anesthesia with epidural	29	13	
Duration of anesthesia (min)	470 (410–585)	580 (500–640)	0.084
Duration of surgery (min)	345 (261–445)	443 (350–499)	0.077
Duration of AXC (min)	66 (55–88)	58 (46–95)	0.624
Fluids infusion (ml)	5560 (4300–6750)	5560 (4100–6890)	0.924
Estimated blood loss (ml)	2620 (1620–3926)	2343 (2179–3835)	0.765
Operative and postoperative details			
Mechanical ventilation, *n* (%)	0 (0)	2 (29)	
Length of hospital stay (days)	19 (16–28)	20 (17–24)	0.917
RRT required upon discharge	0 (0)	0 (0)	
In-hospital death	0 (0)	0 (0)	

Data are expressed as median (interquartile range) or number (%)

*AKI* acute kidney injury, *BMI* body mass index, *ASA* American Society of Anesthesiologists, *ACE inhibitor* angiotensin-converting enzyme inhibitor, *ARB* angiotensin II receptor blocker, *NSAIDs* nonsteroidal anti-inflammatory drugs,* Cr* creatinine, *SCr* serum creatinine, *eGFR* estimated glomerular filtration rate, *RRT* renal replacement therapy, *AXC* aortic cross-clamping

^a^eGFR was calculated according to the Japanese coefficient-modified Chronic Kidney Disease Epidemiology Collaboration equation: eGFR = 194 x (creatinine)^−1.094^ × (age)^−0.287^ × (0.739 if female)^22^

**Fig. 2:**
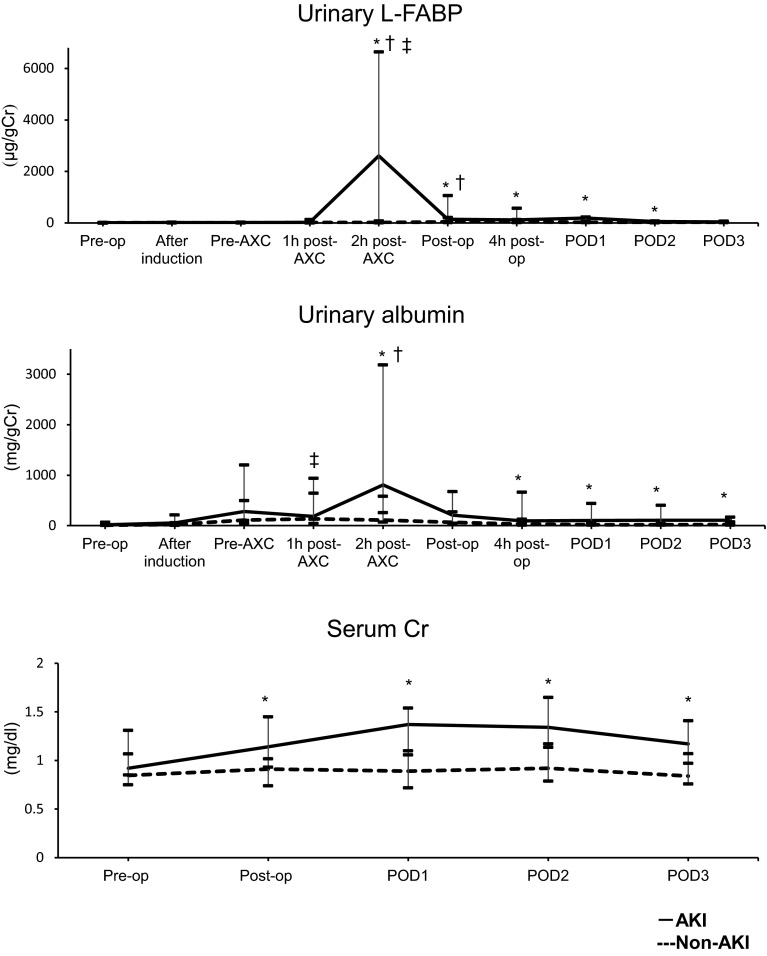
Changes in urinary L-FABP, urinary albumin and SCr in OR. Acute kidney injury (AKI; *solid line*) and non-AKI groups (*dashed line*). *Pre*-*op* preoperative value, *After induction* value after induction of anesthesia, *AXC* value after aorta cross-clamping, *Post*-*op* immediate postoperative value, *4 h post*-*op* value 4 h after operation, *POD* postoperative day, *L*-*FABP* liver-type fatty-acid-binding protein, *SCr* serum creatinine, *OR* open repair *P < 0.05 vs. the non-AKI group at the same time point; ^†^ *P* < 0.05 vs. the respective preoperative level in the same (AKI) group; ^‡^ *P* < 0.05 vs. the respective preoperative level in the same (non-AKI) group

In open-repair patients in whom AKI developed, urinary L-FABP levels were significantly increased to their maximum by 2 h after AXC (*P* = 0.007). They decreased gradually thereafter to POD 3 (Fig. 2). SCr levels were significantly increased immediately after surgery and on PODs 1, 2, and 3 (*P* = 0.010, 0.001, 0.001, and 0.002, respectively; Fig. 2). Urinary albumin levels were significantly increased 2 h after AXC, 4 h after surgery, and on PODs 1, 2, and 3 (*P* = 0.009, 0.008, 0.002, 0.002, and 0.010, respectively; Fig. 2). Results of multivariate logistic regression analysis for both AKI and non-AKI groups showed urinary L-FABP at 2 h post-AXC and SCr at POD2 to be predictors of postoperative AKI (OR 1.58, CI 1.13–2.21, *P* = 0.007; OR 64.0, CI 4.03–1016.2, *P* = 0.003; Table 6). The cutoff urinary L-FABP level at 2 h post-AXC was 173.0 μg/g Cr (Table 7).

**Table 6:** Results of multivariate logistic regression analyses for AKI in the open-repair group

Variable	Multivariate analysis		
	OR	95 % CI	*P* value
Sex	1.13	0.00–969.6	0.971
Age	1.51	0.95–2.42	0.085
Ischemic heart disease	0.78	0.44–1.38	0.389
Urinary L-FABP 2 h post-AXC	1.58	1.13–2.21	0.007
Urinary L-FABP postoperation	0.56	0.12–2.63	0.103
Urinary L-FABP 4 h postoperation	0.50	0.06–3.97	0.515
SCr postoperation	0.00	0.00–87.0	0.103
SCr POD1	13.4	0.00–1.15	0.824
SCr POD2	64.0	4.03–1016.2	0.003
Urinary albumin (10 mg/g Cr) 2 h postAXC	1.00	1.00–1.00	0.402

*AKI* acute kidney injury, *OR* odds ratio, *CI* confidence interval, *L*-*FABP* liver-type fatty-acid-binding protein,* Cr* creatinine, *SCr* serum creatinine, *POD* postoperative day

**Table 7:** Urinary L-FABP levels predictive of AKI in the open-repair study

Time point	Cutoff value (μg/g Cr)	Sensitivity	Specificity	PPV	NPV
Preoperation	12.7	0.25	0.81	0.12	0.91
After anesthesia induction	9.9	0.42	0.69	0.12	0.92
Pre-AXC	5.6	0.83	0.42	0.13	0.96
1 h post-AXC	37.2	0.50	0.79	0.20	0.94
2 h post-AXC	173.0	0.67	0.83	0.30	0.96
Postoperation	32.3	0.92	0.54	0.17	0.98
4 h postoperation	348.5	0.50	0.92	0.40	0.95
POD1	112.1	0.62	0.83	0.28	0.95
POD2	48.6	0.54	0.83	0.25	0.95
POD3	19.5	0.85	0.61	0.19	0.97

*L*-*FABP* liver-type fatty-acid-binding protein, *AKI* acute kidney injury, *AXC* aorta cross-clamping, *PPV* positive predictive value, *NPV* negative predictive value, *POD* postoperative day,* Cr* creatinine

Suprarenal AXC was applied in eight patients who underwent open repair; AKI developed in five (63 %), and peak urinary L-FABP concentration was 8410 μg/g Cr (6050–10,995 μg/g Cr). Infrarenal AXC was applied in the other five AKI patients (15 %) who underwent open repair; peak urinary L-FABP concentration in these patients was 90 μg/g Cr (25–212 μg/g Cr).

### ROC analysis

The biomarker with the largest AUC for predicting AKI onset was urinary L-FABP at 2 h after AXC, at the end of surgery, and 4 h after surgery; AUCs were 0.77, 0.75, and 0.76, respectively (Table 8). We determined urinary L-FABP cutoff values at different time points for EVAR and open repair, as shown in Table 7.

**Table 8:** AUC vs. time in the open-repair group

	Pre-operation	After induction	Pre-AXC	1 h post-AXC	2 h post-AXC	Postoperative	4 h postoperative	POD1	POD2	POD3
Urinary L-FABP	0.48 (0.29–0.68)	0.53 (0.34–0.72)	0.55 (0.36–0.73)	0.58 (0.36–0.79)	0.77 (0.58–0.95)	0.75 (0.59–0.91)	0.76 (0.60–0.92)	0.73 (0.54–0.91)	0.72 (0.53–0.90)	0.69 (0.50–0.88)
Urinary albumin	0.64 (0.45–0.86)	0.71 (0.53–0.88)	0.66 (0.48–0.84)	0.57 (0.38–0.77)	0.78 (0.61–0.95)	0.59 (0.38–0.80)	0.76 (0.60–0.91)	0.81 (0.68–0.94)	0.80 (0.66–0.94)	0.75 (0.60–0.90)
SCr	0.61 (0.41–0.81)	–	–	–	–	0.75 (0.58–0.93)	-	0.82 (0.68–0.95)	0.82 (0.66–0.97)	0.81 (0.66–0.96)

Data are 95% confidence intervals)

*AUC* area under the curve, *After induction* after induction of anesthesia, *Postoperative* immediately after surgery, *POD* postoperative day, *AXC* aorta cross-clamping, *L-FABP* liver-type fatty-acid-binding protein, *SCr* serum creatinine

### Differences in patient characteristics and surgical outcomes between EVAR and open repair

Median age of the open-repair patients was 69 years (IQR 65–75 years) and that of the EVAR patients was 78 years (IQR 74–83 years). The median hospital stay of the open-repair patients was 19 days (IQR 16–25 days) and 13 days (IQR 12–15 days) for EVAR patients. Preoperative ischemic heart disease was significantly more prevalent in the open-repair than in the EVAR group (*P* = 0.030). The incidence of AKI was greater in the open-repair than in the EVAR group (31.0 vs. 9.5 %, respectively), increase in urinary L-FABP in open-repair patients was greater than in EVAR patients, and peak urinary L-FABP level occurred earlier in the open-repair group than in the EVAR group. Hospital stays were significantly longer for open-repair patients than for EVAR patients (*P* < 0.001).

## Discussion

The most important results of this study were that preoperative urinary L-FABP was a good predictor of AKI after EVAR, and 2 h post-AXC urinary L-FABP was a good predictor of AKI after open repair. Our data show that urinary L-FABP is a useful biomarker for early AKI detection after abdominal aortic aneurysm repair, whether EVAR or open repair. In patients in whom AKI developed after either procedure, the rise in urinary L-FABP occurred earlier than the rise in SCr. Ueta et al. reported AKI-associated increases in SCr, neutrophil gelatinase-associated lipocalin (NGAL), and L-FABP 2–6 h after EVAR [23]. They also found urinary Cr-corrected NGAL (NGAL/Cr) to be the best predictive marker for AKI [23]. Because AKI occurred only in their thoracic EVAR (TEVAR) group, the TEVAR procedure did not appear to affect renal blood flow differently from the EVAR procedure. Because our study used EVAR, we postulated that the cutoff urinary L-FABP level would be lower in the Ueta et al. study than in our study. Mori et al. observed that low levels of urinary L-FABP during hypothermic surgery for thoracic aortic aneurysm repair preceded the development of AKI [24]. Although urinary L-FABP was 62.1 ng/mg Cr in their AKI group, it was 1130 ng/mg Cr in their non-AKI group after termination of deep hypothermic circulatory arrest. They proposed that urinary L-FABP plays a role in kidney protection [24]. However, while urinary L-FABP levels were higher in their non-AKI group than in their AKI group, levels ranged more widely in the latter group. In addition, if Mori et al. had measured urinary L-FABP at several time points after surgery rather than only before and after cardiopulmonary bypass, their study results may have been different. Recently, Parr et al. reported that urinary L-FABP, but not urinary NGAL or urinary kidney-injury-molecule 1 (KIM-1), predicted poor AKI outcomes [25]. Our finding that urinary L-FABP increases earlier than SCr after AKI agrees with this latter report [25].

Hypoxic events, such as ischemic–reperfusion injury, cause the release of L-FABP from proximal tubular epithelial cells, correlating with the severity of renal injury; hence, urinary L-FABP level increases immediately after tubular damage. Because of low reuptake, L-FABP from the proximal renal tubules is the main source of urinary L-FABP; serum L-FABP does not increase after injury [26, 27]. Furthermore, Nakamura et al. demonstrated that serum L-FABP levels do not reflect urinary L-FABP levels in patients with sepsis [27].

Mori et al. reported that the difference in the patterns of urinary NGAL increase and urinary L-FABP increase after cardiac surgery results from differences in the mechanism of urinary secretion [28]. NGAL is filtered by glomeruli and reabsorbed by proximal tubules, with only 0.1–0.2 % remaining in the urine [28]. In the AKI setting, various stresses increase NGAL in the circulation through neutrophil activation, and the increased amount of NGAL is filtered in glomeruli. Some NGAL molecules are reabsorbed by the damaged proximal tubules, whereas others are excreted. Therefore, increased urinary NGAL is due mainly to impaired renal reabsorption, [29], and it takes longer for the NGAL levels than for urinary L-FABP levels to increase.

Furthermore, in patients with urinary tract infection (UTI), median urinary angiotensinogen (AGT) levels were significantly increased, but urinary proteins NGAL, L-FABP, N-acetyl-beta-D glucosaminidase (NAG) beta 2-microglobulin (BMG), serum AGT, and creatinine levels did not differ significantly between groups [30]. This report showed that urinary L-FABP did not increase in UTIs. To the contrary, urinary NGAL was significantly increased in patients with UTI compared with that in healthy controls. Increased urinary NGAL indicates the presence of inflammatory processes in the urinary tract of adults [31] and is not a specific biomarker of AKI.

Urinary L-FABP increased earlier in our patients who underwent open repair than in those who underwent EVAR. This difference can be attributed to differences in renal tubular insult associated with EVAR and open repair. Abdominal AXC in open repair decreases and causes a shift in renal blood flow [3, 4], which may in turn cause a significant reduction in proximal tubular blood flow and lead to tubular epithelial cell hypoxia. Such hypoxia promotes L-FABP secretion into the urine. The decrease in renal blood flow and shift in the distribution of intrarenal blood flow occurs quickly after AXC. Proximal tubular ischemic–reperfusion injury causes AKI during the period of open repair. AXC decreases renal blood flow, and renal ischemia induces endothelial dysfunction and decreases production of vasodilatory substances such as nitric oxide. After ischemia, blood flow decreased to 60 % of preischemic levels in superficial cortex and to 16 % in the outer medulla, but it increased to 125 % of control values in the inner medulla [5]. This decrease in blood flow to the outer medulla diminishes oxygen and nutrient delivery to tubules in this region, thus increasing the risk of cell injury [5]. Contrast-induced nephropathy (CIN) is the main cause of AKI in the context of EVAR [32]. Itoh et al. reported that contrast medium causes apoptosis of renal tubular cells [33]; Geenen et al. speculated that contrast medium led to tubular necrosis in CIN [13].

In addition, the EVAR procedure may induce thrombosis and embolism, leading to AKI. Urinary L-FABP increased earlier in our patients who underwent open repair than in patients who underwent EVAR. Furthermore, the increase in urinary L-FABP was less with EVAR than with open repair, indicating that the degree of injury in EVAR was less. The hemodynamic change seems to be greater during open repair than during EVAR, and this change causes further deterioration of tubular blood flow and leads to severe renal damage. The increase in urinary L-FABP in open repair involving suprarenal AXC was greater than that in open repair involving infrarenal AXC. This difference indicates that suprarenal AXC causes more severe renal damage than that caused by infrarenal AXC.

Results of our study should be considered in light of its limitations. For instance, the observed AKI was not severe in most cases; it was stage 2 in all but one patient. Moreover, the elevation in urinary L-FABP was transient; it did not persist after surgery, leaving some doubt as to the degree of kidney injury that had occurred. We also did not take into account any differences in anesthesia. Patients who underwent open repair received sevoflurane with remifentanil and epidural anesthesia; patients who underwent EVAR received sevoflurane with remifentanil but not epidural anesthesia. Furthermore, various vasoactive drugs were administered during open repair but not during EVAR. It is possible that either of the anesthesia methods affected the renal circulation. However, Peyton et al. [34] reported no significant effect of epidural anesthesia on renal complications after major abdominal surgery. Therefore, we believe that our anesthesia methods had little, if any, effect on our study outcomes.

In conclusion, our study highlights urinary L-FABP as a sensitive biomarker of AKI in patients treated with abdominal aortic aneurysm repair. Preoperative urinary L-FABP can predict postoperative AKI, especially in patients treated with EVAR. Urinary L-FABP at 2 h post-AXC can predict postoperative AKI, in patients treated with open repair. In light of these results, we can expect perioperative monitoring of urinary L-FABP to become standard practice for AKI detection in patients treated with abdominal aortic aneurysm repair.
